# Case of Cyanosis

**Published:** 1855-10

**Authors:** Edmund Delany

**Affiliations:** Fond du Lac, Wisconsin


### ART. III.— *Case of Cyanosis.* By Edmund Delany, M. D., Fond du Lac, Wisconsin.

The case of cyanosis, reported by Prof. White, and published in the number of your Journal for July, induces me to send you the following case, which has a very close resemblance to his, but is more remarkable for the length of its continuance:

Martha Hope, aged 12 years, died in this city on the 15th of July, 1853. I had known her for a period of four years, as a neighbor's child. She seemed as active as most girls of her age, but tired more readily than her mates, and was thought asthmatic. There was a blueness, added to her dark complexion, that led to a suspicion of some irregularity in the circulation, and my former partner, Dr. Blodgett, now of Green Bay, and myself, had examined her chest both by the ear and stethoscope, and were of the opinion that the *foramen ovale* had remained unclosed. There was but a *single* sound audible, on the most careful and repeated examinations, and that always a loud *blowing* sound. This was so distinct as to be not only an annoyance to herself, but to the other children with whom she slept.

The only serious attack of disease she suffered, was the whooping cough, which showed no unusual severity for the first three weeks, and no medical aid was sought. At that stage she went out strawberrying, and waded through brooks, from which exposure she had a severe attack of pneumonia, which terminated fatally after four weeks. I only saw her, in consultation, during her illness, but feeling a considerable interest in the anatomical question at issue, obtained leave to make a *post-mortem* examination. In company with Drs. Walker and Galloway, of this place, I made the autopsy twenty-four hours after death. Found purulent infiltration of the entire mass of both lungs. On detaching the heart, and removing it so as to make a careful examination at our leisure, we found the aorta standing directly over the septum ventriculorum, and the passage from the left ventricle closed by *two* semilunar valves,—that from the right ventricle by *one.* A pencil, or a finger passed readily from one ventricle to the other *through the aorta.*

On the closest scrutiny, no vestige of a *foramen ovale*, or a *ductus arte-*

*riosus* could be discovered. The walls of the right ventricle, however, *were of exactly the same thickness as those of the left.* A dense fibrinous concretion was found attached to the *chordæ tendinea* of the tricuspid valve, three lines in diameter and nine inches long, lying in the pulmonary artery and the right branch of that vessel.

Thus venous and arterial blood must have been distributed in nearly equal proportions to the whole body; but a partial compensation appears to have been provided in the increased muscular power of the right ventricle, giving it more than the normal ratio to the pulmonary circulation.

I have been unable to find any notice of a similar malformation of the heart; and as an exceedingly rare, if not unique case, deem it worthy of publication.

